# Prognosis-Based Early Intervention Strategies to Resolve Exacerbation and Progressive Lung Function Decline in Cystic Fibrosis

**DOI:** 10.3390/jpm11020096

**Published:** 2021-02-03

**Authors:** Neeraj Vij

**Affiliations:** 1 Precision Theranostics Inc., Baltimore, MD 21202, USA; nvij@precisiontheranostic.com or nvij@vijbiotech.com or nvij@jhmi.edu; Tel.: +1-240-623-0757; 2 VIJ Biotech, Baltimore, MD 21202, USA; 3 Department of Pediatrics & Pulmonary Medicine, The Johns Hopkins University School of Medicine, Baltimore, MD 21287, USA

**Keywords:** CFTR, cystic fibrosis, prognosis, lung, precision medicine, airway, therapeutics, COPD, asthma, obstructive lung diseases, exacerbation

## Abstract

Cystic fibrosis (CF) is a genetic disease caused by a mutation(s) in the CF transmembrane regulator (CFTR), where progressive decline in lung function due to recurring exacerbations is a major cause of mortality. The initiation of chronic obstructive lung disease in CF involves inflammation and exacerbations, leading to mucus obstruction and lung function decline. Even though clinical management of CF lung disease has prolonged survival, exacerbation and age-related lung function decline remain a challenge for controlling the progressive lung disease. The key to the resolution of progressive lung disease is prognosis-based early therapeutic intervention; thus, the development of novel diagnostics and prognostic biomarkers for predicting exacerbation and lung function decline will allow optimal management of the lung disease. Hence, the development of real-time lung function diagnostics such as forced oscillation technique (FOT), impulse oscillometry system (IOS), and electrical impedance tomography (EIT), and novel prognosis-based intervention strategies for controlling the progression of chronic obstructive lung disease will fulfill a significant unmet need for CF patients. Early detection of CF lung inflammation and exacerbations with the timely resolution will not only prolong survival and reduce mortality but also improve quality of life while reducing significant health care costs due to recurring hospitalizations.

## 1. Introduction

Cystic fibrosis (CF) is an autosomal recessive monogenic disease caused by a mutation(s) in the CF transmembrane conductance regulator (*cftr*), a cAMP-regulated chloride channel gene, discovered in 1989 [1,2,3]. One of the primary functions of the membrane CF transmembrane regulator (CFTR) protein is to transport chloride ions across the epithelial cells of the respiratory and intestinal tracts [3], where impaired CFTR function in CF due to *cftr* mutation(s) results in an ion transport dysfunction [1,3]. This leads to the concentration of mucus in the CF airway due to impaired mucociliary clearance mechanisms, which promotes recurring infections, and initiation of the pathogenesis of chronic inflammatory and obstructive lung disease [4,5,6,7,8]. In addition to its classical role in ion transport, CFTR is known to mediate bacterial phagocytosis and regulate innate immune responses, where its genetic or acquired dysfunction promotes recurring infections and chronic exacerbations [4,9,10,11,12,13,14,15,16]. Moreover, CFTR is known to be expressed on epithelial cell membranes and lipid rafts [17], regulating tight junction formation, ceramide accumulation, and inflammatory-apoptotic responses [10,18,19,20,21,22].

Even though the median life expectancy of CF subjects has significantly increased over the last few decades because of improved quality of care and novel therapeutic development, it remains significantly lower than non-CF healthy subjects [23]. Both recurring exacerbations and chronic inflammation induce lung function decline in CF subjects from a young age, which becomes worse over time, even with available therapeutics, owing to age-related lung function deterioration [24]. Hence, prognosis-based early intervention and constant monitoring of lung disease progression are vital for further prolonging survival and improving quality of life among CF patients.

The classical diagnostic tools for monitoring the progression of CF and the efficacy of therapeutic intervention include sweat chloride analysis, nasal potential difference (NPD), and pulmonary function test (PFT) [25]. Other emerging diagnostics include forced oscillation technique (FOT) and impulse oscillometry (IOS) devices, such as Vyntus^TM^ IOS (Vyaire Medical) & MasterScreen IOS (Vyaire Medical) and TremoFlo (Thorasys Thoracic Medical Systems Inc., Montreal, QC, Canada), which evaluate the impedance in obstructive lung diseases that induce CF, COPD, and asthma [26]. FOT requires reactance standardization and a skilled user and is subject to improvement with the coupling of FOT with artificial intelligence (AI) modalities. Also, the advent of the lung clearance index (LCI) and functional lung imaging modalities (CT, MRI, PET, and X-ray fluoroscopy) have provided better and efficient ways of quantifying changes in regional CF lung function [27,28,29,30,31,32]. Moreover, recent developments in electrical impedance tomography (EIT) allow non-invasive, radiation-free bedside regional lung function analysis by quantifying expiratory time constants and real-time changes in ventilation and perfusion [33,34,35,36]. Currently, Drager’s PulmoVista^R^ 500 and Swisstom AG’s Swisstom BB^2^ are two devices that are commercially available for bedside lung EIT.

The classical sputum and bronchoalveolar lavage fluid (BALF) prognostic biomarkers of CF include elevated levels of IL-8 chemokine and other cytokines, such as interleukin (IL)-10, IL-4, and tumor necrosis factor (TNF)-α [4,10,37]. Besides, increased neutrophil levels are measured by quantifying neutrophil elastase (NE) or myeloperoxidase (MPO) [4,11,38,39,40,41] for quantifying changes in the CF lung inflammatory state. Elevated levels of NFκB and metalloproteinases (MMP) (i.e., MMP-2 and MMP-9) are also known to be associated with increased NE activity and CF lung disease [37,42]. Exacerbation biomarkers include quantification of MUC5AB and MUC5AC degradation and increased sialyation during infection, as well as elevated granulocyte-macrophage colony-stimulating factor (GM-CSF) and neutrophil protein calprotectin [37]. Moreover, serum IL-8 and TNF-α levels are similarly elevated during exacerbation as well as constitutively, as compared with healthy or non-CF subjects. C-reactive protein (CRP), serum amyloid A, leukocyte RNA, and AAT:CD16b complex [11,43,44,45] are other novel biomarkers that have been evaluated for CF prognosis. NPD, PFT, and sweat chloride remain gold standard outcome measure of therapeutic efficacy and/or disease progression; however, the effectiveness of currently available biomarkers is associated with one or more CF outcomes with low effect sizes, making them unacceptable for clinical application [46] or predicting disease or its severity. Thus, despite significant advances in CF care, one of the key challenges and unmet needs is still to identify effective prognosis-based early intervention strategies to resolve exacerbations and progressive lung function decline in CF to both prolong survival and allow a better quality of life.

## 2. Sweat Chloride Test and Nasal Potential Difference Measurements in Cystic Fibrosis

The classical prognostic indicators of CFTR function have been sweat chloride test and nasal potential difference (NPD) measurements [25,47,48]. The sweat chloride concentration and CFTR function (NPD) have not only been used for diagnosis and therapeutic effectiveness but also to predict long-term prognosis. Briefly, sweat chloride has been the gold standard of CF diagnosis, where quantitative pilocarpine iontophoresis is used to evaluate sweat chloride concentration. The procedure uses stimulation of sweating on the forearm or thigh using pilocarpine, which is collected on filter paper, gauze (Gibson-Cooke), or microbore tubing (Macroduct), followed by the quantification of chloride concentration (mmol/L) in the sweat sample [25]. Below 30 mmol/L chloride concentration is considered as suggestive of unlikely CF, while 30–59 mmol/L is intermediate likelihood, and 60 mmol/L or higher is indicative of CF and requires CFTR gene mutation analysis and/or CFTR function via NPD [25].

NPD, although not commercially available, is used at a few CF centers in CFTR function diagnostics and as a measure of early therapeutic effects in clinical trials. NPD quantifies the potential difference using an electrode placed on the nasal surface, followed by a bath with a series of solutions that includes Ringer’s saline solution (baseline NPD), amiloride (blocks sodium channels), chloride-free solution, and isoproterenol, which stimulates CFTR function. The NPD is quantified using two electrodes, with one placed on the nasal mucosa and another one inserted under the skin for the reference. Compared with normal subjects, CF subjects have more negative NPD baseline, larger inhibition of NPD in presence of amiloride, and little or no change in NPD with chloride-free and isoproterenol-containing solutions [48,49,50].

In recent clinical trials of CFTR correctors and potentiators, decreased sweat chloride concentrations and increased CFTR function via NPD measurement have been correlated with improvement in the lung function and (body mass index) BMI of CF subjects. The sweat chloride test and NPD are not direct prognostic biomarkers for evaluating the impact of exacerbation(s) and changes in the lung function. Thus, highlighting the need to develop novel prognostics to monitor lung function decline and disease progression for evaluating the impact of exacerbation(s) and therapeutics on progression of CF lung disease.

## 3. Pulmonary Function Tests for Cystic Fibrosis Lung Disease Progression

Spirometry-based pulmonary function testing quantifying FEV1 (forced expiratory volume in 1 s) has been a gold standard for evaluating CF lung disease progression as well as the impact of exacerbations and intervention(s) on lung function [32,37,51]. Although it is readily available and inexpensive, it only allows longitudinal changes in airflow obstruction over the years to be quantified. Briefly, PFT quantifies the lung’s ability to exchange oxygen and carbon dioxide or the amount of air the patient can inhale and exhale by performing a series of inspirations and expirations on the mouthpiece of the spirometer [37]. FEV1 is the amount of air a patient forces out in the first second of exhalation, where less than 80% of the predicted value is considered abnormal, and 10% below the subject’s baseline is indicative of lung function decline requiring intervention. Typically, CF patients will have a 1–2% FEV1 baseline drop each year as a result of progressive lung tissue damage, and it is crucial to stop this progressive lung function decline to increase the average median survival of CF subjects [37]. One of the key challenges of spirometry-based PFT is patient compliance, especially in young children or chronically sick and elderly subjects. In addition to spirometry-based PFT, diffusion capacity is a test that quantifies the ability of airspaces to allow oxygen to diffuse across the interstitium into the bloodstream and is commonly known as DLCO (diffusing capacity for carbon monoxide). Thus, DLCO quantifies the ability of the lungs to transfer gas from inhaled air to red blood cells (RBC) in pulmonary capillaries [52]. DLCO has been shown to have a significant correlation with measures of airflow limitation (forced vital capacity, FVC, FEV1/FVC) in adult CF subjects [53], although it has no scope in detecting subtle or early changes. Similarly, homebased spirometry monitoring of CF patients was able to detect more exacerbations than standard of care, but it was unable to limit the decline in lung function [54] because of its inability to detect early subtle or regional changes in lung function.

To summarize, spirometry-based FEV1/PFT can certainly quantify significant or progressive lung function decline but is unable to identify subtle or regional changes in lung function, hence making its utility limited for evaluating the immediate effect of recurring exacerbations or therapeutic interventions. This limits FEV1′s ability as an early prognostic indicator or ability to quantify regional real-time subtle changes in lung function. 

## 4. Lung Clearance Index for Cystic Fibrosis Airway Disease Progression

To address the limitations of FEV1 in detecting early lung disease progression or subtle lung function decline, the lung clearance index (LCI), a measurement of multiple breath washouts (MBW) of inert gas, is now used as a better prognostic indicator of CF progression [27,28]. Briefly, LCI is a numerical value derived from a MBW dataset, where it represents the number of breaths needed to reduce a tracer or inert gas to a predefined concentration [32,37]. Tracer gases commonly used in this test include nitrogen (N_2_) and sulfur hexafluoride (SF_6_), and variables that impact LCI results not only include the choice of the tracer gas but also the equipment used and body position (upright or supine). Typically, increased equipment dead space results in higher LCI, which requires the use of facemasks for infants and preschoolers and a nose clip and mouthpiece for the subjects.

LCI has been demonstrated to not only detect early lung disease but also better quantify the impact of exacerbations or interventions, and it correlates with high-resolution computed tomography (HRCT) findings over the standard of care, FEV1 [32]. LCI also correlates with early CF inflammation and predicts the risk of pulmonary exacerbation, but longitudinal data are not yet available to determine its effectiveness in chronic or progressive lung disease [37]. In fact, its utility in patients with advanced lung disease (percent predicted FEV1 < 60%) is limited due to profound ventilation heterogeneity and extended wash-in and wash-out periods. However, in pediatric subjects and patients with compliance issues or inability to perform spirometry tests, LCI serves as an optimal diagnostic tool with the ability to quantify early or acute prognostic changes. LCI requires only passive tidal breathing and can be used with infants and young children without sedation or need for mechanical manipulation. Thus, LCI serves as an optimal prognostic tool for detecting early lung disease and tracking its progression, but it is not useful for advanced stages of CF lung disease.

## 5. Functional Lung Imaging Modalities for Evaluating Cystic Fibrosis Lung Disease Progression

Chest and sinus CT is typically used in CF to detect early lung disease/bronchiectasis and mucus-plugging, respectively [30,37]. Moreover, HRCT is used for detecting the impact of exacerbations and lung disease progression, where CT-based functional analysis using 3D reconstruction of the airway and pulmonary vasculature allows the quantification of airflow obstruction, changes in airway size, and/or blood volume in pulmonary vasculature [34,55,56,57]. Similarly, advances in magnetic resonance imaging (MRI)-based functional imaging and X-ray-fluoroscopy-based ventilation and lung heterogeneity analysis allow the quantification of regional lung function changes to predict prognosis and efficacy of interventions [28,29,30,34,55,57]. These modalities have a varying capability for predicting prognosis, exacerbation, and/or effectiveness of an intervention. HRCT is considered most sensitive for quantifying changes in airway thickness and the effect and extent of exacerbation and bronchiectasis [32]. MRI (typically using hyperpolarized helium) and fluorodeoxyglucose positron emission tomography (FDG-PET) are considered more sensitive in detecting acute changes than HRCT, which is more powerful for evaluating chronic disease with structural changes [30,34,55].

With advances in functional imaging software and the advent of artificial intelligence (AI) analysis and classical radiological scoring methods for evaluating CF lung disease severity, the quantification of changes in airway and vasculature structure, airflow, mucus-pugging, or obstruction will allow early prognosis as well as monitoring of disease progression for prognosis-based interventions. However, the challenge with radiological imaging remains a certain level of risk with each of these modalities as well as the inability to monitor real-time changes on the bedside in order to evaluate the relative effectiveness of an intervention. Hence, advances in methods for non-invasive, bedside, and real-time regional lung function analysis and/or quantification of other prognostic indicators are needed to push forward precision medicine for avoiding gradual lung function decline and decreasing the impact of recurring exacerbations in the CF population.

## 6. Force and Impulse Oscillometry Measurements for Regional Lung Function Analysis

The innovation of novel medical devices for the forced oscillation technique (FOT), such as TremoFlo (Thorasys), and impulse oscillometry systems (IOS), such as Vyntus IOS, CareFusion IOS, and MasterScreen IOS, allows the quantification of impedance in obstructive lung diseases for regional lung function analysis in infants and younger children [26]. Briefly, FOT and IOS measure airway resistance, similar to the body plethysmography, a technique that has been effectively used to measure lung function in infants and young CF subjects, who are unable to perform spirometry. FOT non-invasively evaluates lung mechanics using the relationship between pressure and flow, where waveform, forced oscillations (for FOT), and impulse oscillations (for IOS) are used to quantify the mechanical impedance of the respiratory system or the airway [26,58,59,60,61]. One of the advantages of these systems over classical spirometry is that they do not require patient compliance, allowing its application with infants, young children, and chronically ill subjects [26].

The underlying technique involves superimposed sound waves over normal tidal breathing, where a disturbance in externally applied waves is used to passively measure changes in pressure and flow, and output parameters include the resistance of airflow or impedance [26,58,59,60,61]. The entropy of impedance can also help differentiate the frequency and onset of exacerbation as well as the efficacy of an intervention. FOT- and IOS-based measurements require reactance standardization for variation that can now be automated using AI technology for quantitative assessment of early changes in lung function and prognosis-based intervention. Besides, this diagnostic technique allows quantification of bronchodilator response and bronchoprovocation testing. In addition, quantitative output of early changes in lung function is far better than the current gold standard, spirometry. The resistance or impedance measurements provide an assessment of regional inhomogeneity and lung periphery, with minimal patient cooperation [60]. Thus, further AI-based FOT/IOS clinical development and standardization will allow widespread application over conventional spirometry-based global assessment of changes in lung function, where subtle localized and early lung dysfunction is often missed in disease states such as CF.

## 7. Electrical Impedance Tomography for Non-Invasive Regional Lung Function Analysis

Electrical impedance tomography (EIT) uses an electric current to non-invasively assess the distribution of alternating current conductivity in the lungs as a method to quantify changes in ventilation (V) and perfusion (Q) by continuous bedside monitoring. In addition to radiation-free bedside functional lung imaging for V/Q analysis, EIT can provide quantification of regional time constants [33,34,35,36]. The continuous and functional evaluation of the patient’s lungs or respiratory status is the keystone for critical care and can provide real-time assessment of the therapeutic or pulmonary clinical interventions.

Briefly, EIT uses continuous or repeated quantification of surface voltages or impedance, generated from a rotating injection of low-intensity and high-frequency alternating current through the electrodes around the patient’s thorax [33,35]. These impedance measurements allow the quantification of the volume of gas or air entering upon each inspiration in the region of interest, forming a relative image that is compared with a reference or baseline [35]. EIT utilizes detection of recruitment, de-recruitment, silent spaces, or poorly ventilated lung units and respiratory time constants in real-time, which can allow the adjustment of mechanical ventilation in critical care subjects as well as evaluation of early regional lung function changes for timely intervention [36]. 

EIT can also provide an estimate of local lung perfusion in parallel to ventilation signal mapping for quantification of regional V/Q ratios and is available in real-time and on the bedside for effective and precise intervention [33]. Thus, commercially available novel EIT diagnostic devices such as PulmoVista^R^ 500 and Swisstom BB^2^ allow real-time or bedside non-invasive visualization of regional air distribution within the lungs for prognosis-based interventions. It also allows monitoring of the therapeutic or clinical intervention efficacy, with data available as an imaging modality as well as quantitative numbers. EIT generates global and regional impedance waveforms, regional ventilation distribution (RVD), and regional compliance changes, where pulmonary function can be monitored up to a 24 h period at a time on the patient’s bedside, using a silicone belt with 16 incorporated electrodes that can be easily placed around patient’s chest and connected to the EIT device for real-time monitoring of regional lung function changes [33,35].

## 8. Prognostic Biomarkers of Cystic Fibrosis Lung Disease

As discussed above, classical sputum and bronchoalveolar lavage fluid (BALF) prognostic biomarkers of CF include elevated levels of IL-8 chemokine and other cytokines, such as IL-10, IL-4, and TNF-alpha, as well as increased neutrophil elastase (NE) or myeloperoxidase (MPO) activity [2,4,10,11,13,43,62,63]. Elevated levels of NFkB and metalloproteinases (MMP), namely MMP-2 and MMP-9, are associated with increased NE activity and CF lung disease pathogenesis and progression [11,42,64]. The biomarkers of CF exacerbation include MUC5AB and MUC5AC degradation, due to increased sialyation during infection, as well increased serum/blood or BALF levels of granulocyte-macrophage colony-stimulating factor (GM-CSF), IL-8, TNF-alpha, and neutrophil protein calprotectin, as compared to healthy subjects [2,4,37,39,44,45,63]. Several other novel biomarkers that have been evaluated for CF prognosis include c-reactive protein (CRP), serum amyloid A, leukocyte RNA, and AAT:CD16b complex [11,43,44,45]. NPD, PFT, and sweat chloride continue to serve as the gold standard diagnostics as FDA-accepted outcome measures of therapeutic efficacy and CF lung disease progression, as no prognostic biomarker till date has gone through quantification standards as a surrogate endpoint of clinical efficacy in CF [65,66].

**Figure 1:**
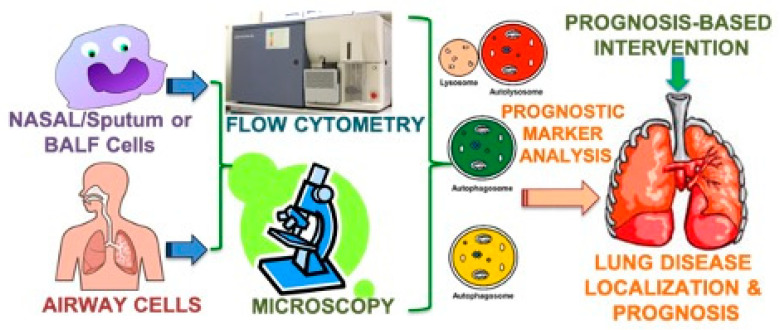
Schematic showing prognostic marker analysis for personalized cystic fibrosis (CF) lung disease intervention. CF transmembrane conductance regulator (CFTR) and prognostic marker(s); membrane, cytosolic or aggresomal expression and localization in induced-sputum/nasal or BALF/airway cell samples using high throughput flow cytometry and/or microscopy based computational analysis for prognosis-based targeted therapeutic intervention.

In addition to biomarkers of inflammation in CF [65,66], microbial biomarkers of acute and chronic infection, including standard colony-forming units, viral load, microbial metabolites, and molecular diagnostics of infection, are commonly used in the clinical management of exacerbations [4,24,67,68,69], with limited scope for prognosis-based early intervention. Hence, the clinical development and validation of novel prognostic-biomarkers that permit early detection or prediction of an exacerbation may allow the resolution of progressive lung disease and maintenance of stable lung function in CF subjects. Moreover, delivering on the full potential of precision-medicine novel non-invasive prognostic indicators of CF lung disease progression is needed for effective and timely intervention as discussed below. Thus, surrogate biomarkers that provide prognostic or predictive information regarding CF lung disease or its response to treatment will help personalize therapeutics, where early prognostic changes can be detected and quantified using CF patient’s cells (nasal, induced sputum, BALF) and blood (serum, plasma) samples as shown in Figure 1. In addition, these lab or clinical validation prognostics can be developed as point-of-care (POC) or home-based tests using visual or compatible POC readers for early identification of exacerbation to allow timely intervention and avoid recurring hospitalizations.

## 9. Prognosis-Based Intervention Strategies for Cystic Fibrosis Exacerbations and Lung Disease

Personalized medicine has been at the forefront of research on CF, where the underlying cause is a genetic mutation in CFTR, and thus recent therapies have primarily focused on correction, potentiation, and read-through approaches to restore the functional CFTR protein. Gene therapy including CRISPR/Cas9 technology can allow full correction of the genetic mutation, which remains the ultimate goal for the CF cure; however, there are various challenges with these approaches that need to be overcome before gene therapy or editing can be used for the treatment of CF. It certainly remains difficult to deliver the CFTR gene or the gene-editing tools to the target lung epithelial cells for stable transduction or correction of the mutation [70,71,72,73]. As a part of the PROMISE trial, CF researchers are currently evaluating the efficacy of Trikafta^TM^ (Elexacaftor/Tezacaftor/Ivacaftor) on improving lung function and controlling infection, inflammation, and mucus obstruction [52,70,72,73,74]. Likewise, the GOAL and PROSPECT clinical trials are also evaluating the CF therapeutics impact on infection and inflammation, although there remains a significant gap in designing therapeutics capable of resolving CF exacerbations and lung function decline [52,70,72,73,74,75].

Hence, screening and designing tailored therapies focused on prognosis-based restoration of lung function decline or circumvention of the impact of recurring exacerbations on lung function will not only prolong survival and reduce repeated hospitalization costs but will also improve quality of life for patients with CF [76]. Thus, the basis of these therapeutic interventions or preventive strategies remains to be a novel prognostic indicator of CF exacerbation and lung function decline. As discussed above, current CF diagnostics and prognostics are focused on identifying CF mutations and quantifying changes in infection, inflammation, and lung function [66,70,72,73,75]. As we move forward with making further progress on personalized medicine for CF healthcare, we need to develop prognostic indicators as point-of-care (POC) tests and non-invasive diagnostics that allow early as well as continuous monitoring of CF lung disease status for timely intervention.

Spirometry remains the gold standard outcome measure for CF drug development but may not be ideal for identifying therapies for early prognostic interventions in exacerbation or rapidly declining lung function, as it quantifies global changes but is unable to identify subtle and regional changes in CF lungs. Although there has been some progress in functional lung imaging modalities, they have shortcomings in the longitudinal assessment of therapeutic efficacy as well as in repeated or real-time monitoring of CF lung disease progression due to repeated radiation, contrast agent, and tracer gas exposure risks. The comparative utility and advantages of currently available and emerging CF diagnostics are summarized in Table 1 and Table 2, respectively. The recent developments in FOT, IOS, and EIT diagnostic devices circumvent these shortcomings but require further standardization and potential AI-based analysis to quantify narrow subtle changes in regional lung function. These novel devices can fulfill the unmet need regarding both therapeutic development and prognosis-based bedside intervention. However, the key to early intervention for recurring exacerbation, which is the prime factor of lung function decline, is designing prognostic POC and home care tests that are readily available for early intervention to avoid repeated hospitalizations and declining lung health.

## 10. Perspective

Even though the average life expectancy continues to improve for CF patients given the significant improvements in CF care, diagnostics, and therapeutics, recurring exacerbations and declining lung function remain a significant issue where prognosis-based early intervention(s) may help reduce repeated hospitalization costs, improve quality of life, and deliver on the promise of normal life expectancy for CF patients. We have a variety of diagnostics and analytical scoring systems for calculating the severity of CF [25,28,77] but still lack precise prognostic indicators of exacerbation and declining lung function. Thus, a prognosis-based early intervention strategy for exacerbation(s) and progressive lung function decline remains a significant unmet need in CF.

Besides, a novel outcome measure for predicting exacerbation(s) as well as lung function decline is required for the development of tailored therapies for an effective early resolution of exacerbation, circumventing a significant impact on lung function. Moreover, risk stratification as well as predictive outcome measures will allow timely prognosis-based intervention and resolution to further improve CF patients’ survival [37,43]. Thus, the multidisciplinary approach of CF health care will be greatly transformed with next-generation personalized care solutions such as novel prognostic indicators for maintaining optimal lung function and preventing CF lung disease progression. Noteworthy is that the clinical development and bedside translation of tailored prognosis-based CF therapies and interventions requires the concerted effort of innovators, entrepreneurs, regulators, payers, and policymakers. 

The current CF prognostic strategies involve a combination of multiple biomarkers of disease, imaging modalities, including PFT, NPD, and sweat chloride, where therapeutic screening has relied on the improvement of CFTR’s ion transport function (NPD, sweat chloride) followed by a subsequent assessment of inflammatory or infection biomarkers, PFT, and/or lung imaging [25,55]. Thus, the complexity of outcome measures and the lack of real-time assessment of lung disease progression limit the ability to prevent exacerbations and gradual lung function decline leading to eventual transplant or mortality. To address this shortcoming, precision theranostics are emerging as a strategy with the potential for delivering on the real-time assessment of therapeutic interventions that will revolutionize both primary CF health care and targeted therapeutic development. Briefly, theranostic systems possess a prognosis- or diagnosis-based therapeutic release that allows real-time assessment of functional and therapeutic efficacy [78]. The application of theranostics in CF includes targeted cell delivery, nanoparticle-based contrast agents for imaging, real-time assessment of CFTR-therapeutics and lung function, and predicting or tracking exacerbations. Theranostics may target epithelial and inflammatory cells in vivo, as we recently described [38,78,79,80,81], or utilize ex vivo patient samples or stem cells as cell therapy or biologics [82,83,84,85]. 

In summary, the development of predictive or prognostic indicators of CF exacerbations impacting lung function as well as a real-time assessment of lung disease progression will lead to more efficient prognosis-based early therapeutic and clinical interventions, improving the survival and quality of life of CF subjects.

## Figures and Tables

**Table 1:** Currently used and emerging cystic fibrosis & lung disease diagnostic comparison. PFT: Pulmonary Function Test, LCI: Lung Clearance Index, POC ID: Point of Care Infectious Disease, FOT: Forced Oscillation Technique, IOS: Impulse Oscillometry System, EIT: Electrical Impedance Tomography.

Cystic Fibrosis & Lung Diagnostics	Lung Function/ Disease Analysis	Compatibility with Intervention	Predicts Initiation of Exacerbation	Setting	Associated Risks
Lung Imaging	Yes	Average	No	Radiology	High
PFT/Spirometry	Yes	Low	No	PFT Lab	No
LCI	Yes	Average	No	Bedside	No
Sweat Chloride	No	Low	No	Lab/Clinic	No
POC ID	No	High for ID	Yes	POC	No
FOT/IOS/EIT	Yes	High	No	Bedside	Minimal
Prognostics	Yes	Excellent	Yes	POC/Lab	No

**Table 2:** Advantage of respiratory diagnostics for cystic fibrosis. PFT: Pulmonary Function Test, PET: Positron Emission Tomography, MRI: Magnetic Resonance Imaging, CT: Computed Tomography, LCI: Lung Clearance Index, POC ID: Point of Care Infectious Disease, FOT: Forced Oscillation Technique, IOS: Impulse Oscillometry System, EIT: Electrical Impedance Tomography.

Lung Diagnostic Advantage for CF	Cost ofDiagnostic/Test	Resolution/ Specificity	Level of Accuracy	Patient Compliance	Time to Complete
Thoracic X-Ray	Low	Low	Low	Not Required	5 min
Thoracic PET/MRI	Very High	Moderate	Moderate	Required	60–120 min
Thoracic CT Scans	High	High	High	Required	20 min
PFT/Spirometry	Moderate	Low	Moderate	Patient Dependent	30–60 min
POC ID	Moderate	High	High	Not Required	15 min-72 h
LCI	Moderate	High	High	Required	15–40 min
Sweat Chloride	Low	Average	Average	Not Required	24–48 h
FOT/IOS/EIT	Moderate	High	High	Not Required	Real Time/Continuous
Prognostics	Moderate	Very High	High	Not Required	15–40 min

## Data Availability

Not applicable for review article.
